# Asymptomatic recurrence detection and cost-effectiveness in urothelial carcinoma

**DOI:** 10.1007/s12032-018-1152-1

**Published:** 2018-05-09

**Authors:** Hiromichi Iwamura, Shingo Hatakeyama, Makoto Sato, Chikara Ohyama

**Affiliations:** 1 Department of Urology, Hirosaki University Graduate School of Medicine, 5 Zaifu-cho, Hirosak, 036-8562 Japan; 2 Department of Urology, Tohoku Medical and Pharmaceutical University, 1-15-1 Fukumuro, Sendai, 983-8536 Japan

**Keywords:** Cystectomy, Nephroureterectomy, Urothelial carcinoma, Recurrence, Surveillance, Symptomatic, Cost-effectiveness

## Abstract

For the management of muscle-invasive bladder cancer or upper tract urothelial carcinoma, the set guidelines recommend regular surveillance after radical cystectomy or radical nephroureterectomy. However, the prognostic benefit of regular oncological surveillance remains controversial in the absence of prospective studies although several retrospective studies with relatively large sample sizes have demonstrated the association between asymptomatic recurrence and better oncological outcomes. Seven out of eight studies reported that patients diagnosed with symptomatic recurrence showed significantly poorer prognosis in comparison to those diagnosed with asymptomatic recurrence. However, potential lead-time and length-time biases prevent the determination of any benefit of regular surveillance. In addition, an optimal surveillance protocol has yet to be established because conventional pathology-based protocols cannot identify the heterogenetic tumor biology of urothelial carcinoma, such as rapid- or slow-growing form of the disease. Several studies suggest that conventional pathology-based surveillance resulted in reduced cost-effectiveness. Recurrence risk-score stratified surveillance protocol including clinical and pathological factors may improve cost-effectiveness. The establishment of optimal risk stratification and surveillance strategies are required to improve the efficacy of regular oncological surveillance. Well-planned prospective studies are necessary to address the prognostic benefit of regular oncological surveillance and shared decision making.

## Introduction

Radical cystectomy (RC) and radical nephroureterectomy (RNU) are standard therapy for muscle-invasive bladder cancer (MIBC) and upper tract urothelial carcinoma (UTUC), respectively [1–8]. Because recurrence after radical surgery for urothelial carcinoma leads to poor prognosis, early detection followed by salvage therapies is generally considered important [5, 9–12]. Whether regular surveillance after RC for MIBC or RNU for UTUC could improve oncological outcomes remains controversial due to surveillance-related biases and the lack of prospective studies. Additionally, most studies fail to demonstrate any survival benefit of regular surveillance in colorectal cancer [13], breast cancer [14], endometrial cancer [15], or lung cancer [16]. Similarly, debates continue on whether regular oncological surveillance to detect asymptomatic recurrence after RC or RNU improves patient survival [17, 18]. Furthermore, cost-effectiveness represents another important factor to consider for regular surveillance. Although larger number of screens increase the medical cost, less screening could translate into missing a chance for therapy. Several guidelines recommend regular oncological surveillance [5, 10–12]; however, these guidelines do not address medical cost. Currently, no established cost-effective surveillance protocol after RC or RNU is available [5, 19, 20], and only few studies have investigated a cost-effective surveillance protocol after RC [21] and RNU [22]. This review summarizes the current evidences of the benefit and cost-effectiveness of regular oncological surveillance to detect recurrence after RC and RNU.

## Asymptomatic recurrence and oncological outcomes

### MIBC

**Fig. 1:**
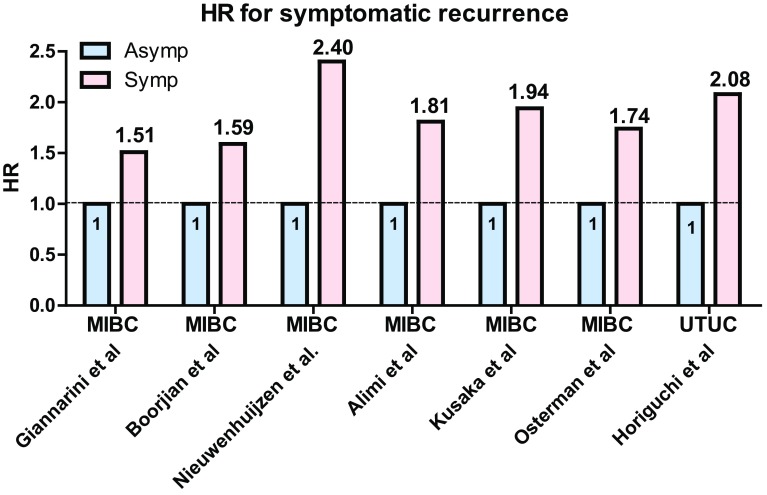
Summary of previous studies aimed at investigating the impact of detecting asymptomatic recurrence during regular surveillance after radical cystectomy. Multivariate Cox regression analysis shows that patients diagnosed with symptomatic recurrence during regular surveillance have significantly worse prognosis compared to those diagnosed with asymptomatic recurrence

Despite advances in surgical techniques and neoadjuvant/adjuvant chemotherapies for MIBC, approximately 38–49% of patients experience disease recurrence within 10 years after RC [2, 9, 23]. Once metastatic disease has occurred, its prognosis is dismal due to limited and transient benefit of salvage therapy, and the median survival after recurrence is approximately 12–14 months [24]. Therefore, the benefit of regular oncological follow-up to detect asymptomatic recurrence has been questioned. To date, seven retrospective studies have investigated the impact of detecting asymptomatic recurrence at regular surveillance after RC [17, 25–30] (Table 1; Fig. 1). Of the seven studies, only one by Volkmer et al. [25] reported no survival benefit of detecting asymptomatic recurrence and concluded that symptom-guided follow-up may provide similar results at lower cost. However, the authors excluded secondary urothelial recurrence from all postoperative recurrences, potentially underestimating the frequency of asymptomatic recurrences and lowering the oncological benefit of regular surveillance. Furthermore, they concluded the lack of benefit of detecting asymptomatic recurrence based on only a log-rank test without multivariate analysis. Conversely, the recent six studies using multivariate Cox regression analysis demonstrated the benefit of detecting asymptomatic recurrence [17, 26–30]. Giannarini et al. [26] reviewed 479 patients who underwent RC with orthotopic ileal neobladder reconstruction and showed that patients diagnosed with symptomatic recurrence during regular surveillance had significantly worse cancer-specific survival (CSS) and overall survival (OS) compared to those diagnosed with asymptomatic recurrence [CSS: hazard ratio (HR) 1.54; *P* = 0.013 and OS: HR 1.51; *P* = 0.015] (Fig. 1). Boorjian et al. [27] retrospectively investigated 1599 patients who underwent RC and demonstrated that symptomatic recurrence was an independent risk factor for worse OS (HR 1.59; *P* = 0.0001) (Fig. 1). Nieuwenhuijzen et al. [28] also reviewed 343 patients treated with RC and reported that symptomatic recurrence was adversely associated with CSS (HR 2.40; *P* = 0.013) (Fig. 1). Alimi et al. [29] reported the prognostic disadvantage of symptomatic recurrence in a series of 331 patients who underwent RC (CSS: HR 1.81; *P* = 0.049) (Fig. 1). Similarly, Osterman et al. [30] reviewed 463 patients who underwent RC and showed worse OS in the symptomatic group than in the asymptomatic group (HR 1.74; *P* < 0.05) (Fig. 1). Furthermore, Kusaka et al. [17] evaluated the impact of symptomatic recurrence after RC in 581 patients. Their results showed that 53% of patients presented with symptoms at recurrence after RC and found that patients with symptomatic recurrence had a significantly worse prognosis than with asymptomatic recurrence. Patients with an asymptomatic recurrence frequently experienced lymph node recurrence, whereas symptomatic patients exhibited larger number of local pelvic recurrence cases. In addition, they used an inverse probability of treatment weighting (IPTW) strategy to remove the effects of confounding factors. The IPTW-adjusted Cox regression analysis performs reweighting of affected and unaffected groups to emulate a propensity score-matched population [31] in order to evaluate the impact of symptomatic recurrence on prognosis. The IPTW-adjusted analysis showed that symptomatic recurrence was an independent risk factor for OS after RC (HR 1.94; *P* < 0.001) and OS after recurrence (HR 2.18; *P* < 0.001). From these retrospective studies, asymptomatic recurrence detection is suggested as an independent prognostic factor after RC (Fig. 1). Taken together, these results suggest a clinical benefit of regular surveillance after RC; however, prospective studies are needed to confirm its potential benefit.

**Table 1:** Summary of previous studies for prognostic risk of symptomatic recurrence after radical cystectomy or radical nephroureterectomy

Authors (year)	No. of patients	No. of patients with recurrence (%)	Symptomatic versus asymptomatic (%)	Prognostic risk of symptomatic recurrence	Analysis
Bladder cancer					
Volkmer et al. (2009)	1,270	444 (49%)	65% versus 35%	No (not significant in OS)	Univariate, log rank test
Giannarini et al. (2010)	479	174 (36%)	50% versus 50%	Yes (HR 1.51, *P* = 0.015 in OS)	Multivariate Cox regression analysis
Boorjian et al. (2011)	1,599	606 (38%)	77% versus 23%	Yes (HR 1.59, *P* = 0.0001 in OS)	Multivariate Cox regression analysis
Nieuwenhuijzen et al. (2013)	343	158 (46%)	64% versus 36%	Yes (HR 2.40, *P* = 0.013 in CSS)	Multivariate Cox regression analysis
Alimi et al. (2016)	331	160 (49%)	81% versus 19%	Yes (HR 1.81, *P* = 0.040 in CSS)	Multivariate Cox regression analysis
Kusaka et al. (2017)	581	175 (30%)	53% versus 47%	Yes (HR 1.94, P < 0.001 in OS)	IPTW-adjusted multivariate Cox regression analysis
Osterman et al. (2017)	463	197 (43%)	54% versus 36%	Yes (HR 1.74, P < 0.05 in OS)	Multivariate Cox regression analysis
UTUC					
Horiguchi et al. (2017)	415	108 (26%)	43% versus 57%	Yes (HR 2.08, *P* = 0.009 in OS)	IPTW-adjusted multivariate Cox regression analysis

*OS* overall survival, *CSS* cancer-specific survival, *HR* hazard ratio, *IPTW* inverse probability of treatment weighting

### UTUC

The standard treatment for non-metastatic UTUC is RNU with bladder cuff excision [5, 7, 8, 11], and regular surveillance after RNU is considered necessary. Although the rationale for regular surveillance is a better prognosis based on earlier recurrence detection, evidence proving the benefit of detecting asymptomatic recurrence after RNU is scarce. To date, only one study has investigated the clinical benefit of regular surveillance after RNU [18] (Table 1; Fig. 1). Horiguchi et al. [18] retrospectively reviewed 415 patients treated with RNU for UTUC at four hospitals in Japan. Their cohort included 108 (26%) patients with tumor recurrence. The number of patients with asymptomatic and symptomatic recurrences was 62/108 (57%) and 46/108 (43%) patients. Their results showed that patients with symptomatic recurrence had a significantly worse overall survival than those with asymptomatic recurrence [18]. These findings are consistent with those of our previous study on symptomatic recurrence after RC [17]. Recurrence-free survival, CSS after RNU, and OS after recurrence were significantly longer in patients in the asymptomatic group than in those in the symptomatic group. IPTW-adjusted multivariate Cox regression analysis showed that symptomatic recurrence was an independent risk factor for OS after RNU (HR 1.75; *P* = 0.040) and OS after recurrence (HR 2.08; *P* = 0.009) (Fig. 1). Therefore, regular oncological surveillance for detecting asymptomatic recurrence after RNU potentially improves prognosis. Although a prospective randomized study is required, accumulation of retrospective studies is also needed because UTUC is a relatively rare disease [5, 32].

## Impact of lead-time bias on outcomes

On regarding the oncological benefit of regular surveillance for asymptomatic recurrence detection, lead-time bias must be considered. Lead-time bias means that the survival duration after asymptomatic recurrence may be overestimated because surveillance-detected recurrence is generally detected earlier than symptomatic recurrence. Although there are no prospective studies to resolve lead-time bias in regular follow-up after RC or RNU, Osterman et al. [30] retrospectively attempted to account for lead-time bias in patients who underwent RC. They showed that symptomatic recurrence was diagnosed 1.7 months before asymptomatic recurrence; nevertheless, median survival after symptomatic recurrence was 8.2 month shorter than after asymptomatic recurrence. These results suggest that detecting asymptomatic recurrence after RC represents an oncological benefit, which cannot be explained by lead-time bias.

## Risk factors for symptomatic recurrence

**Fig. 2:**
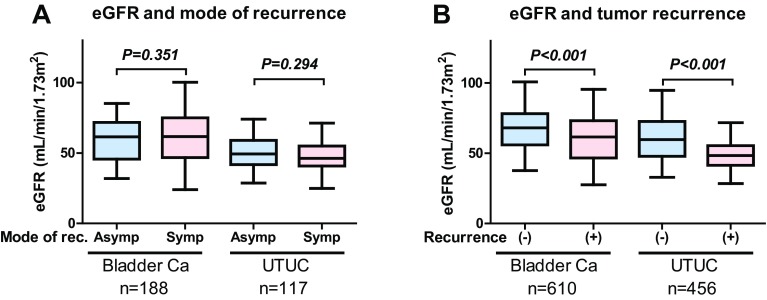
Association between renal function and mode of recurrence. Preoperative eGFR is not significantly different between patients with asymptomatic and symptomatic recurrence (**a**). Preoperative eGFR is significantly lower in patients with recurrence than in those without recurrence (**b**)

**Fig. 3:**
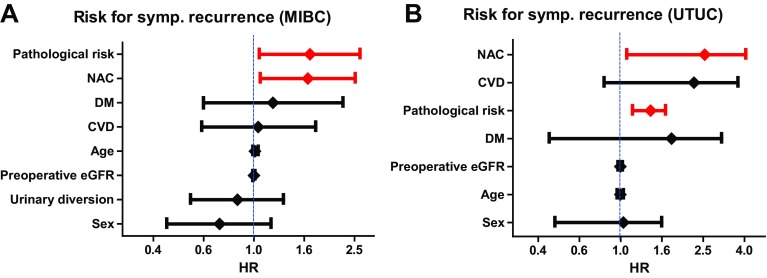
Potential risk factors for symptomatic recurrence. Multivariate Cox regression analysis shows that neoadjuvant chemotherapy (NAC) and the presence of pathological risk (pT3–4, LVI, or pN+) are independent factors for symptomatic recurrence in MIBC (**a**) and UTUC (**b**)

**Fig. 4:**
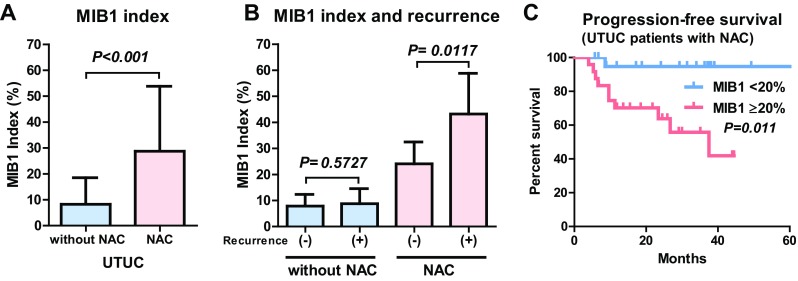
Association between MIB1 index and neoadjuvant chemotherapy (NAC) in patients with UTUC. MIB1 index is significantly higher in patients with UTUC treated with NAC (21%; IQR 6.9–44%) than in those without NAC (3.3%; IQR 1.9–12%) (**a**). The median MIB1 index is significantly higher in patients with NAC with relapse than in those without relapse (16% vs. 39%) but did not differ in those without NAC (4.0% vs. 2.4%) (**b**). MIB1 index > 20% was significantly associated with poor progression-free survival in patients with UTUC treated with NAC (**C**)

Symptomatic recurrence represents a failure of regular surveillance when it occurs through the regular follow-up examinations. Identifying the risk factors for symptomatic recurrence could contribute to improving the surveillance protocol, resulting in better oncological outcomes after RC or RNU. Recently, Anan et al. [6] retrospectively investigated the risk factors for symptomatic recurrence in 581patients after RC, including 100 symptomatic and 75 asymptomatic recurrences. Symptomatic recurrences were significantly frequent within 24 months after RC. Multivariate Cox regression analysis identified lymphovascular invasion (LVI) as an independent risk factor for symptomatic recurrence. In addition, several previous studies [33–39] suggested the association between preoperative severe renal insufficiency and symptomatic recurrence in patients with urothelial carcinoma. Because no study has investigated the relationship between renal insufficiency and symptomatic recurrence, we analyzed the impact of preoperative eGFR status on mode of recurrence in the same cohort reported by Momota et al. (MIBC, *n* = 610; UTUC, *n* = 456) [39]. Our analysis suggests that preoperative eGFR was not significantly different between patients with asymptomatic and symptomatic recurrence (Fig. 2a) although it was significantly lower in patients with recurrence compared to patients without recurrence (Fig. 2b). In addition, multivariate Cox regression analysis showed that eGFR was not an independent factor for symptomatic recurrence in both MIBC and UTUC. These results implied that preoperative eGFR could not identify the high-risk patients with symptomatic recurrence after radical cystectomy or nephroureterectomy. In contrast, the use of neoadjuvant chemotherapy (NAC) and pathological risk associated (pT3–4, LVI, or pN+) were identified as independent factors for symptomatic recurrence in both MIBC and UTUC (Table 2; Fig. 3a, b). These results suggest a significant impact of NAC on symptomatic recurrence in urothelial carcinoma, although previous studies suggested significant risk reduction of NAC use for tumor recurrence in both MIBC [40–42] and UTUC [43–45]. One possible reason is selection of the malignant clone during NAC for urothelial carcinoma. Recent study suggested a potential association between the MIB1 index (immunostaining for Ki67), a proliferation marker, and NAC use [43]. Hosogoe et al. reported the MIB1 index was significantly higher in patients with UTUC with NAC compared to those without NAC [43]. The median MIB1 index was significantly higher in the patients with NAC (21%; IQR 6.9–44%) than in those without NAC (3.3%; IQR 1.9–12%) (Fig. 4a). In addition, the median MIB1 index was significantly higher in patients with relapse than in those without relapse in the patients with NAC (16% vs. 39%) but did not differ in those without NAC (4.0% vs. 2.4%) (Fig. 4b). The authors suggested that MIB1 index > 20% was significantly associated with poor progression-free survival in patients with UTUC with NAC (Fig. 4c). Therefore, selection of the malignant clone during NAC may impact tumor aggressiveness and resulted in symptomatic recurrence, whereas the total number of recurrence events decreased after NAC [43]. Although further studies are necessary to elucidate the relationship between symptomatic recurrence and NAC use, close attention is recommended when patients with urothelial carcinoma have LVI and higher MIB1 index after RC or RNU.

**Table 2:** Multivariate Cox regression analysis for symptomatic recurrence after radical cystectomy (*n* = 610) or radical nephroureterectomy (*n* = 456)

	Risk factor	*P* value	HR	95% CI
MIBC				
Age	Continuous	0.247	1.01	0.99–1.04
Sex	Male	0.193	0.73	0.45–1.17
CVD	Positive	0.880	1.04	0.62–1.76
DM	Positive	0.595	1.19	0.63–2.26
Preoperative eGFR	Continuous	0.765	1.00	0.99–1.01
NAC	Underwent	0.025	1.64	1.06–2.53
Urinary diversion	Ileal neobladder	0.483	0.86	0.56–1.31
Pathological risk	pT3–4, LVI, or pN+	0.029	1.67	1.05–2.64
UTUC				
Age	Continuous	0.838	1.00	0.96–1.03
Sex	Male	0.645	0.87	0.48–1.58
CVD	Positive	0.138	1.76	0.83–3.70
DM	Positive	0.734	1.18	0.45–3.08
Preoperative eGFR	Continuous	0.857	1.00	0.97–1.02
NAC	Underwent	0.031	2.08	1.07–4.04
Pathological risk	pT3–4, LVI, or pN+	0.001	1.37	1.14–1.65

*CVD* cardiovascular disease, *DM* diabetes mellitus, *eGFR* estimated glomerular filtration rate, *NAC* neoadjuvant chemotherapy

Although detection of asymptomatic recurrence is associated with improved patient survival [17, 18, 26–30], the underlying reason may be linked to identifying the difference between rapid- and slow-growing tumors (i.e., length-time bias). In fact, the clinical characteristics of symptomatic and asymptomatic recurrence are different. As a previous study suggested, symptomatic recurrences are more frequent in local pelvis and/or bone. However, asymptomatic recurrence are more frequently lymph node recurrences [17]. Tumor progression that is missed during routine follow-up suggests a biological heterogeneity between rapid- and slow-growing tumors that cannot be detected by a conventional pathological examination. In addition to clinical or pathological information, novel biomarkers predicting the malignant potential of tumors are required for a better understanding of tumor biology. In recent times, the wide-spreading technology of gene testing may allow to predict organs that are likely to metastasize. Genome-based molecular is one of the potential biomarkers for phenotype classification [46–49]. The basal type of MIBC is associated with worse disease-specific and OS due to its highly invasive and metastatic potential [46]. This phenotype is also associated with an epithelial–mesenchymal transition and bladder cancer stem-cell biomarkers [50]. Furthermore, the earlier detection of recurrence is pivotal for the possibility to use immune-checkpoint inhibitors [51, 52] that potentially provide long-term survival for selected patients. Novel biomarkers predicting the malignant potential of tumors can applied for surveillance schedules in future.

## Cost-effectiveness in regular follow-up after surgery

**Fig. 5:**
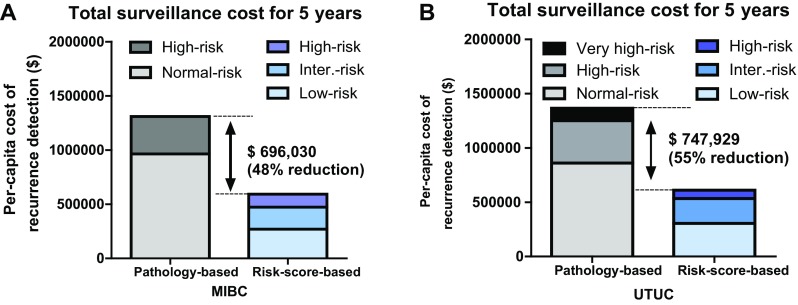
Comparison of total surveillance cost for 5 years between pathology-based and risk-score-based protocols. The estimated medical cost differences reached $696,030 (48% reduction) in MIBC after RC (**a**). The estimated medical cost differences reached $747,929 (55% reduction) in UTUC after RNU (**b**)

Expensive follow-up cost is justified only when surveillance leads to improved patient survival. Asymptomatic recurrence identification implies effectiveness of regular surveillance; however, there are no clear guidelines on how to appropriately follow-up patients after RC [20] or RNU [5, 19, 53]. Based on the principle that most recurrences after RC or RNU tend to occur within 2 years [9, 21, 22], several follow-up protocols have been suggested by guidelines [5, 10–12, 53] based on the low level of evidence, and these protocols are mainly stratified by pathological stage without considering the heterogeneity in patients who underwent RC or RNU. With more screening, asymptomatic recurrence can be detected earlier; however, cost will increase accordingly. A few studies have investigated the cost-effectiveness of surveillance protocols [21, 22, 54]. Vemana et al. [54] calculated the surveillance cost for 24 months after RC using the Surveillance, Epidemiology, and End Results data base. The actual follow-up cost per patient for 24 months was $1108, and follow-up cost will increase up to 10.6-fold if surveillance were performed based on current established guidelines [9–12, 55]. Kusaka et al. [21] developed a risk-score-stratified surveillance protocol with improved cost-effectiveness after RC. They retrospectively reviewed 581 patients after RC with regular oncological follow-up. The risk-scores were calculated by summing 6 risk factors (LVI+, pN+, ≥ pT3 or surgical margin [SM]+, preoperative chronic kidney disease [CKD]+, cardiovascular disease+, and non-neobladder) that were independently associated with recurrence-free survival by multivariate analysis. Patients were classified into 3 groups; low-risk (0–1), intermediate-risk (2–3), and high-risk (4–6) groups (Table 3) [21]. Based on this risk stratification, the authors developed a risk-score-based protocol for oncological follow-up and evaluated the per-capita cost of recurrence detection. The estimated per-capita cost of recurrence detection was compared between the risk-score-based protocol and the conventional pathology-based protocol. The risk-score-based protocol led to a dramatic cost reduction compared to the pathology-based protocol. The total estimated 5-year screening cost was 1.9-fold higher with the pathology-based protocol ($1,148,687) than with the risk-score-based protocol ($613,901). Estimated cost differences reached $534,786 per recurrence detected, a suggested 48% reduction in this cohort (Fig. 5a) [21].

**Table 3:** Risk-score-based classification for MIBC

Variable	Status	Risk-score
Cardiovascular disease	Positive	1
Preoperative CKD	Positive	1
Urinary diversion	Non-neobladder	1
Pathological T stage	≥ pT3 or SM+	1
Pathological N stage	pN positive	1
Lymphovascular invasion	Positive	1

Risk-score-based classification	Sum of risk-score
Low-risk	0−1
Intermediate-risk	2−3
High-risk	4−6

*CKD* chronic kidney disease, *SM* surgical margin

With respect to UTUC, only one study has investigated the cost-effectiveness of regular oncological surveillance. Momota et al. [22] developed a risk-score-stratified surveillance protocol with improved cost-effectiveness after RNU. They retrospectively reviewed 426 patients after RNU with regular oncological follow-up. Risk-scores were calculated by summing 7 risk factors (SM+, LVI+, ≥ pT3, preoperative CKD+, cN+ or pN+, hydronephrosis+, and tumor in ureter) that were independently associated with recurrence-free survival by multivariate analysis. Patients were classified into 3 groups; low-risk (0–2), intermediate-risk (3–5), and high-risk (6–12) groups (Table 4) [22]. Based on this risk stratification, they developed a risk-score-based protocol for oncological follow-up and evaluated the per-capita cost of recurrence detection. The estimated per-capita cost of recurrence detection was compared between the risk-score-based protocol and the conventional pathology-based protocol. Their results suggested that the total estimated 5-year surveillance cost was 2.2-fold higher with the pathology-based protocol ($1,365,245) than with the risk-score-based protocol ($617,315). Estimated cost differences reached $747,929 per recurrence detected, a suggested 55% reduction in this cohort (Fig. 5b) [22]. These results suggested a higher cost of the conventional pathology-based protocol and the importance of using surveillance protocols considering clinical factors associated with recurrence after RC or RNU.

**Table 4:** Risk-score-based classification for UTUC

Variable	Status	Risk-score
Tumor in ureter	Positive	1
Hydronephrosis	Positive	1
Lymph node involvement (cN+ or pN+)	Positive	2
Preoperative CKD	Positive	2
Pathological T stage	pT3–4	2
Lymphovascular invasion	Positive	2
Surgical margin	Positive	2

Risk-score-based classification	Sum of risk-score
Low-risk	0−2
Intermediate-risk	3−5
High-risk	6−12

*CKD* chronic kidney disease

## Limitations

The present review has several limitations. Firstly, it is an accumulation of retrospective studies with a small number of patients with recurrence. It is difficult to control all variables including selection bias, the influence of lead-time biases, and other unmeasurable confounding factors. Secondly, results cannot be extrapolated independently to other countries because of the difference in health insurance system. Despite these limitations, this review supports the idea that a cost-effective surveillance protocol following curative surgery would detect recurrence at the early stages of disease. In addition, the detection with asymptomatic recurrence should secure sufficient time to implement a multimodal therapy after relapse. Although a prospective randomized study comparing a symptom-based surveillance would be ideal to clarify the survival benefit of a routine surveillance protocol, a prospective study would be difficult to conduct due to ethical concerns. Therefore, the accumulation of evidences from well-planned retrospective studies is needed to support the clinical benefit of routine surveillance protocol on prognosis and cost-effectiveness.

## Conclusion

Although asymptomatic recurrence detected by regular surveillance potentially results in better oncological outcomes after RC or RNU, the prognostic benefit of regular oncological surveillance remains unclear. The establishment of optimal risk stratification and surveillance strategies are required to improve the efficacy of regular oncological surveillance. Well-planned prospective studies are necessary to address the prognostic benefit of regular oncological surveillance and shared decision making.
